# *Leishmania*-Specific Surface Antigens Show Sub-Genus Sequence Variation and Immune Recognition

**DOI:** 10.1371/journal.pntd.0000829

**Published:** 2010-09-28

**Authors:** Daniel P. Depledge, Lorna M. MacLean, Michael R. Hodgkinson, Barbara A. Smith, Andrew P. Jackson, Saufung Ma, Silvia R. B. Uliana, Deborah F. Smith

**Affiliations:** 1 Centre for Immunology and Infection, Department of Biology, Hull York Medical School, University of York, York, United Kingdom; 2 Wellcome Trust Sanger Institute, Wellcome Trust Genome Campus, Hinxton, United Kingdom; 3 Departamento de Parasitologia, Instituto de Ciências Biomédicas, Universidade de São Paulo, São Paulo, Brazil; Institut Pasteur, France

## Abstract

**Background:**

A family of hydrophilic acylated surface (HASP) proteins, containing extensive and variant amino acid repeats, is expressed at the plasma membrane in infective extracellular (metacyclic) and intracellular (amastigote) stages of Old World *Leishmania* species. While HASPs are antigenic in the host and can induce protective immune responses, the biological functions of these *Leishmania-*specific proteins remain unresolved. Previous genome analysis has suggested that parasites of the sub-genus *Leishmania (Viannia)* have lost HASP genes from their genomes.

**Methods/Principal Findings:**

We have used molecular and cellular methods to analyse HASP expression in New World *Leishmania mexicana* complex species and show that, unlike in *L. majo*r, these proteins are expressed predominantly following differentiation into amastigotes within macrophages. Further genome analysis has revealed that the *L. (Viannia)* species, *L. (V.) braziliensis,* does express HASP-like proteins of low amino acid similarity but with similar biochemical characteristics, from genes present on a region of chromosome 23 that is syntenic with the HASP/SHERP locus in Old World *Leishmania* species and the *L. (L.) mexicana* complex. A related gene is also present in *Leptomonas seymouri* and this may represent the ancestral copy of these *Leishmania*-genus specific sequences. The *L. braziliensis* HASP-like proteins (named the orthologous (o) HASPs) are predominantly expressed on the plasma membrane in amastigotes and are recognised by immune sera taken from 4 out of 6 leishmaniasis patients tested in an endemic region of Brazil. Analysis of the repetitive domains of the oHASPs has shown considerable genetic variation in parasite isolates taken from the same patients, suggesting that antigenic change may play a role in immune recognition of this protein family.

**Conclusions/Significance:**

These findings confirm that antigenic hydrophilic acylated proteins are expressed from genes in the same chromosomal region in species across the genus *Leishmania*. These proteins are surface-exposed on amastigotes (although *L. (L.) major* parasites also express HASPB on the metacyclic plasma membrane). The central repetitive domains of the HASPs are highly variant in their amino acid sequences, both within and between species, consistent with a role in immune recognition in the host.

## Author Summary

Single-celled *Leishmania* parasites, transmitted by sand flies, infect humans and other mammals in many tropical and sub-tropical regions, giving rise to a spectrum of diseases called the leishmaniases. Species of parasite within the *Leishmania* genus can be divided into two groups (referred to as sub-genera) that are separated by up to 100 million years of evolution yet are highly related at the genome level. Our research is focused on identifying gene differences between these sub-genera that may identify proteins that impact on the transmission and pathogenicity of different *Leishmania* species. Here we report the presence of a highly-variant genomic locus (OHL) that was previously described as absent in parasites of the *L. (Viannia)* subgenus (on the basis of lack of key genes) but is present and well-characterised (as the LmcDNA16 locus) in all members of the alternative subgenus, *L. (Leishmania)*. We demonstrate that the proteins encoded within the LmcDNA16 and OHL loci are similar in their structure and surface localisation in mammalian-infective amastigotes, despite significant differences in their DNA sequences. Most importantly, we demonstrate that the OHL locus proteins, like the HASP proteins from the LmcDNA16 locus, contain highly variable amino acid repeats that are antigenic in man and may therefore contribute to future vaccine development.

## Introduction

Kinetoplastid parasites of the genus *Leishmania* cause a diverse spectrum of infectious diseases, the leishmaniases, in tropical and subtropical regions of the world (reviewed in [1]). Mammalian-infective *Leishmania* species are divided into two subgenera, *Leishmania* (*Leishmania*) and *Leishmania* (*Viannia*), that differ in their developmental cycles within the female sandfly vector. Transmission of species of both subgenera from vector to mammalian host requires parasite differentiation into non-replicative flagellated metacyclic promastigotes. These forms are inoculated when a female sandfly takes a blood meal; the parasites enter resident dermal macrophages and transform into replicative amastigotes that can be disseminated to other tissues, often inducing immuno-inflammatory responses and persistent infection. The fate of *Leishmania* amastigotes in the host determines disease type, which can range from cutaneous or mucocutaneous infection to diffuse cutaneous or the potentially fatal visceral leishmaniasis [1].

Comparative sequencing of three *Leishmania* genomes, *L. (L.) major* and *L. (L.) infantum* from the *L. (Leishmania)* sub-genus and *L. (V.) braziliensis* from the *L. (Viannia)* sub-genus, has revealed high conservation of gene content and synteny across the genus [2], [3], [4]. A number of loci show significant variation in size and gene complement between species, however. One example is the GP63 locus, containing tandemly arrayed genes coding for surface glycoproteins that are critical for macrophage invasion and virulence [5], [6]. This locus is present in all three sequenced *Leishmania* species but varies considerably in size and number of genes present. Another example is the LmcDNA16 locus, originally identified on chromosome 23 of *L. (L.) major* [7], [8], [9], [10] but since also found in *L. (L.) donovani* [11], *L. (L.) infantum* [3] and other *L. (Leishmania)* species. This locus is characterised by the presence of two *Leishmania*-specific gene families encoding hydrophilic acylated surface proteins (HASPs; [7], [9], [10], [12], [13]) and small hydrophilic endoplasmic reticulum associated proteins (SHERPs; [14]). The HASPs have conserved N- and C- termini but a sub-set, the HASPBs, possess divergent central domains containing hydrophilic amino acid repeats that exhibit both inter- and intra specific variation in their size and composition [10], [11], [15]. Acylation of the HASPBs involves *N-*terminal myristoylation and palmitoylation, modifications that are required for protein targeting to the parasite plasma membrane [13]. In *L. majo*r, HASPB expression is confined to mammalian-infective stages of the parasite life cycle, the metacyclics and amastigotes. While HASPBs are antigenic in the host [16], [17] and can induce protective immune responses [18], [19], [20], the biological functions of both the HASP and SHERP proteins remain unresolved [7].

To date, while the LmcDNA16 locus has been identified in all *L. (Leishmania)* species analysed, expression and localization of the encoded proteins has not been studied in New World *L. (Leishmania)* species. Here, we present analysis of HASPB expression in two representative sub-species, *L. m. mexicana* and *L. m. amazonensis*. Furthermore, the LmcDNA16 locus has been reported as absent from the published *L. (V.) braziliensis* genome, one of the few chromosomal regions showing strong divergence between sub-genera [3]. Instead, an apparently unrelated region containing several putative genes of unknown coding capacity is found in this position on chromosome 23 [3].

In this paper, we investigate this region further and identify at least two novel but closely-related *L. (V.) braziliensis* genes coding for putatively acylated repeat-containing proteins. These, like the HASPB proteins in *L. (L.) mexicana* but unlike those in *L.major*, are predominantly expressed on the plasma membrane of amastigotes. We name these proteins *orthologous HASPs* (oHASPs) and refer to the locus as the *orthologous HASP locus* (OHL). Sequencing one of these new *L. (V.) braziliensis* genes in clinical isolates taken from Brazilian leishmaniasis patients has identified extensive sequence variation in the amino acid repeat regions, while some but not all sera samples taken from the same patients recognise recombinant protein expressed from the same open reading frame expressed in *E. coli.* These data identify a new molecular marker for *L. (V.) braziliensis* infection and suggest the potential for antigenic change within this class of amastigote proteins.

## Materials and Methods

### Genome sequences and computational analyses

The *L. (L.) major*, *L. (L.) infantum, L. (V.) braziliensis* and *Leptomonas seymouri* genome sequences [21], [22] were obtained from GeneDB (www.genedb.org - [23]) during the period June – September 2009. Comparative alignments of the target loci (and flanking regions) were performed using the BLASTALL program [24] and visualised using the Artemis Comparison Tool [25].

*N*-terminal myristoylation and palmitoylation sites in target sequences were predicted using NMT – The MYR Predictor [26], [27] and CSS-Palm 2.0 [28] with default settings. CLUSTAL alignments were generated for inter- and intra-species analysis of the oHASP protein repetitive regions using the CLUSTALW2 program (default settings) hosted by EBI.

### *Leishmania* species and strains

The *Leishmania* species and strains used in this study are described in Table 1 and include 11 *L. (V.) braziliensis* clinical isolates, provided as genomic DNA by the Leishmaniasis Immunobiology Laboratory, Institute of Tropical Pathology and Public Health, Goiás Federal University (Leishbank - IPTSP/UFG/GO). The identities of species and strains were confirmed using restriction fragment length polymorphism (RFLP) analysis [29]. The clinical isolates were identified as *L. (V.) braziliensis* by PCR-typing with ribosomal DNA and glucose-6-phosphate dehydrogenase/META2 genes as described [30], [31], [32].

**Table 1:** *Leishmania* species and strains used in this study.

Species	Strain	Code	Source
*L. major*	MHOM/IL/80/Friedlin FVI*	-	Smith lab cryobank
*L. infantum*	MCAN/ES/98/LLM-877*	-	
*L. donovani*	MHOM/ET/67/L28/LV9	-	
*L. mexicana*	MYNC/BZ/62/M379	-	
*L. amazonensis*	MHOM/BR/73/M2269	-	
*L. guyanensis*	MHOM/BR/75/M4147	LgM4147-75	
*L. peruviana*	MHOM/PE/90/LCA08	LpLCA08-90	P. Volf, Prague
*L. braziliensis*	MHOM/BR/75/M2904 *	M2904-75	A. Cruz, São Paulo
*L. braziliensis*	MHOM/BR/84/LTB300	LTB300	Smith lab cryobank
*L. braziliensis*	MHOM/BR/2006/GDL^+^	GDL-06	S. Uliana, São Paulo/Leishbank - IPTSP/UFG/GO
*L. braziliensis*	MHOM/BR/2006/HPV^+^	HPV-06	
*L. braziliensis*	MHOM/BR/2003/IMG^+^	IMG-03	
*L. braziliensis*	MHOM/BR/2006/PPS^+^	PPS-06	
*L. braziliensis*	MHOM/BR/2006/TMB^+^	TMB-06	
*L. braziliensis*	MHOM/BR/2006/BES^+^	BES-06	
*L. braziliensis*	MHOM/BR/2005/RPL^+^	RPL-05	
*L. braziliensis*	MHOM/BR/2006/UAF^+^	UAF-06	
*L. braziliensis*	MHOM/BR/2005/WSS^+^	WSS-05	
*L. braziliensis*	MHOM/BR/2006/EFSF^+^	EFSF-06	

These include the reference genome strains* of *L. major, L. infantum and L. braziliensis* [2], [3] plus representative strains of other *Leishmania* species and *L. braziliensis* clinical isolates+. Code, as used to describe strains in Figure 7.

*L. (L.) major*, *L. (V.) braziliensis* and *L. (L.) infantum* parasites were maintained in culture as described [33]. *L. (L.) mexicana* and *L. (L.) amazonensis* parasites were maintained in culture and differentiated according to the method of Bates [34]. *L. (V.) braziliensis* promastigotes and intramacrophage amastigotes were generated and purified as described [33]. In brief, macrophages were incubated with stationary-phase *L. (V.) braziliensis* at a ratio of 1∶10 for 2 hr at 34°C, prior to washing twice with DMEM, replacement with fresh complete DMEM and further incubation for 48 hr at 34°C before amastigote harvesting, using 0.05% saponin and a single density isotonic Percoll gradient.

### DNA extraction and analysis

Genomic DNA from each species and strain was extracted as follows: 5×10^8^ – 5×10^9^ parasites were pelleted by centrifugation (2000 g, 10 min, 4°C) and washed twice with sterile PBS. Pellets were resuspended in 9 ml NET Buffer (0.01 M Tris pH 8.0, 0.05 M EDTA, 0.1 M NaCl) and 1 ml 10% SDS, ribonuclease A (Sigma Aldrich) added to a final concentration of 100 µg/ml and the mixture incubated at 37°C for 30 min. 200 µl proteinase K (20 mg/ml) was added and the mixture incubated at 55°C overnight. Parasite genomic DNA was extracted with phenol-chloroform, washed twice in 70% ethanol, resuspended in TE buffer and stored at 4°C.

PCR primers were designed using the Primer3 web utility [35] with default settings and synthesised by Eurogentec. All primer sequences used are shown in Table S1. PCR amplifications for sequencing and cloning were carried out in either a Peltier PTC-200 Thermocycler (MJ Research) or a TechGene Thermocycler (Techne) using the Kod polymerase (Novagen) in 3-step reactions, according to the manufacturer's instructions. Briefly, the initial denaturing step required a 2 min incubation at 94°C and was followed by 35 reaction cycles (1 cycle = 95°C, 30 sec; 55°C, 10 sec; 72°C, 40 sec) and a final extension step of 40 sec at 72°C.

Southern blotting was carried out as described [7] with DIG-labeled probes and hybridization reagents (Roche) using the manufacturer's protocols. Primers for probe amplification were targeted against the intergenic region within the OHL locus (Table S1). The membrane was exposed to autoradiography film (Amersham Hyperfilm HP) and processed using a XoGraph Compact x4 (XoGraph Imaging Systems).

DNA sequencing of the repeat domains of oHASP genes utilised cloned PCR products amplified with suitable flanking primers (Table S1) and cloned into pGEM-T easy vector. All sequencing was carried out on an Applied Biosystems 3130 sequencer, using T7 forward and Sp6 reverse primers, in the University of York Technology Facility; all data were analysed using Applied Biosystems Sequence Scanner v1.0.

### RNA isolation and analysis

Total RNAs (15 µg per track) from procyclic, metacyclic and amastigotes of *L. (L.) mexicana and L. (L.) amazonensis,* generated by axenic culture [34], were extracted and analysed by formaldehyde denaturing electrophoresis in the presence of commercial RNA markers, prior to blotting and hybridization as described [14]. The radioactive probe used for hybridization, NREP, was an oligo-labelled PCR product generated from the repetitive central domain of the HASPB gene (GenBank: AJ251974.1) using primers NREP1 and NREP2 (Table S1).

*L. (V.) braziliensis* amastigote pellets were resuspended using TRIzol Reagent (Invitrogen) and total RNA extracted according to the manufacturer's instructions. Further purification and quantitative real-time PCR (RT-qPCR) analysis was carried out as described [33]. The data generated were normalised using the constitutively expressed γ-glutamyl cysteine synthetase (LbrM18_V2.1700) [36].

### Protein expression, antibody generation and immunodetection

The *L. mexicana* HASPB open reading frame (ORF) was amplified using the primers LEXP5 and LEXP32 (Table S1) prior to cloning into the *Nde*1site of pET15b and expression in *E. coli* BL21 (DE3) pLysS [14]. His-tagged recombinant protein was purified by affinity chromatography, checked for purity by SDS-PAGE, and used to raise polyclonal antibodies (anti-Lmex HASPB) in rabbits, as described in [14].

The Lbr1110 ORF was PCR-amplified from *L. (V.) braziliensis* genomic DNA (wild-type strain), using the Lb1110 primers (Table S1) and subject to ligation-independent cloning within the University of York HiTel facility (http://www.york.ac.uk/depts/biol/tf/hitel/index.htm). The resulting recombinant plasmid was introduced into *E.coli* Rosetta 2 and expression achieved in auto-induction medium [37] with overnight growth at 30°C.

For protein purification, bacterial cells were resuspended in 70 ml buffer containing 300 mM NaCl, 20 mM sodium phosphate pH 7.4, 20 mM imidazole, protease inhibitors and DNAse I. Lysis was performed by one pass through a continuous flow French Press at 20 kPSi and 4°C. The crude lysate was cleared by centrifugation at 50,000 g for 40 min at 4°C followed by filtration of the supernatant through a 0.8 µm membrane. All purification steps were carried out on an AKTA100 (GE) fitted with a direct loading pump. The lysate was loaded directly onto an equilibrated 1 ml HisTrap column (GE) at a flow rate of 1 ml/min. Following a 10 column volume (CV) wash with buffer A (300 mM NaCl, 20 mM sodium phosphate pH 7.4, 20 mM imidazole), bound proteins were eluted with buffer B (300 mM NaCl, 20 mM sodium phosphate pH 7.4, 0.5 M imidazole) using a gradient of 0–100% B over 10 CV. Fractions of 1 ml were collected and analysed by SDS-PAGE; peak fractions were pooled and concentrated to ∼2 ml. Gel filtration was then performed using a Superdex 75 16/60 column (GE) and PBS buffer at a flow rate of 1 ml/min, collecting 1 ml fractions for SDS-PAGE analysis. Purified protein (final yield, ∼4 mg/L cells) was concentrated, stored at −20°C in PBS containing 25% glycerol and used for polyclonal antibody production in rabbits (Eurogentech).

Antibodies were purified using a 1 ml NHS-activated HP column (GE) coupled with 1 mg recombinant Lbr1110 protein. Following column equilibration with 10 ml binding buffer (20 mM sodium phosphate pH7, 150 mM NaCl), 15 ml rabbit serum was loaded onto the column at 0.3 ml/min. Unbound sample was removed with 5 ml binding buffer and antibody eluted at low pH (in 0.1 M glycine pH2.7, 0.5 M NaCl) in 0.5 ml fractions directly into tubes containing 50 µl 1 M Tris-HCl pH9 for neutralisation and storage.

For immunoblotting, total protein lysates from 2×10^6^ parasites were separated by SDS-PAGE prior to transfer on to PVDF Immobilon P membrane (Millipore), as described [14]. The resulting blots were probed with rabbit anti-Lb1110 (1∶1000), anti-Lmex HASPB (1∶500) and mouse anti-EF1-α (1∶1000; Millipore). Immune complexes were detected by ECL reagents (Amersham Biosciences), with 30 sec exposure times. To detect immune recognition by clinical sera, similar blots were probed with sera from CL patients (1∶300 to 1∶500) and control healthy individuals (also Brazilian), prior to detection with anti-human HRP (1∶5000; Sigma).

For detection by confocal microscopy, antibody-labelling was performed on live parasites, to detect surface Lb1110, and on permeabilised cells, to detect total Lb1110 localisation. 2×10^7^ parasites were collected by centrifugation at 800 g for 10 min, washed and resuspended in 100 µl of 1% fatty acid-free BSA blocking solution (BB International) for 20 min. Live parasites were labelled with rabbit anti-Lb1110 (1∶100) for 30 min at 20°C, then fixed in 4% paraformaldehyde (PFA) before secondary detection with AlexaFluor-488-conjugated goat anti-rabbit IgG (1∶250 in blocking solution; Invitrogen). Labelling was also carried out on permeabilised cells which were first fixed in 4% PFA, washed, then incubated with 0.1% Triton-X100 (Sigma) for 10 min, washed and then incubated in 1% BSA blocking solution for 20 min at 20°C before labelling as above. Parasites were allowed to adhere to polylysine slides (Sigma) for 20 min and coverslips mounted with Vectashield containing DAPI (Vector Laboratories), prior to imaging using a Ziess LSM 510 meta with a Plan-Apochromat 63X/1.4 oil DIC I objective lens. Images were acquired using LSM510 version 3.5 software.

**Figure 1:**
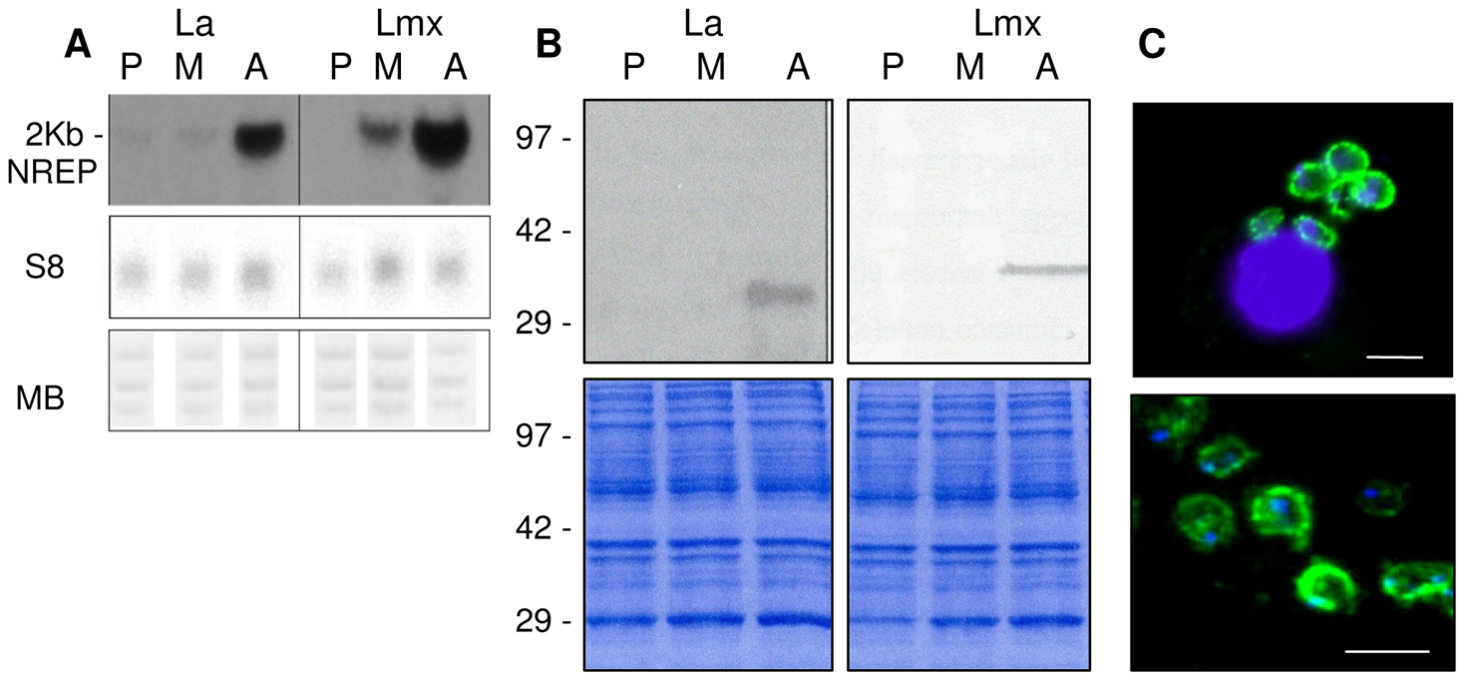
Expression of HASPB genes in *L. (L.) mexicana* and *L. (L.) amazonensis.* A. RNA expression: total RNAs from were size separated in the presence of formaldehyde, blotted and hybridised sequentially with probes specific for the HASPB gene repeat regions (NREP) and the ribosomal S8 gene (S8). The filters were also stained with methylene blue (MB) before hybridisation. The size of the HASPB transcript is shown on the left of the blots. B. Protein expression: total parasite lysates (using 2×10^6^ parasite-equivalents per track) from procyclic (P), metacyclic (M) and axenic amastigotes (A) of *L. (L.) amazonensis* and *L. (L.) mexicana* were analysed by SDS-PAGE, prior to blotting with antibodies raised against recombinant *L.(L.) mexicana* HASPB (top panel). Bottom panel: Coomassie-stained gels prior to blotting; molecular mass markers are shown on the left (kDa). C. Detection of *L. (L.) mexicana* HASPB expression in fixed amastigotes within a macrophage (top panel) and from axenic culture (bottom panel). Immunofluorescence microscopy using the LmxHASPB antibody from B (green) and counterstaining with DAPI (blue) reveals the large macrophage nucleus (top panel) and smaller parasite nuclei and kinetoplasts (in both panels). Size bar: 5 µm.

For detection by epifluorescence microscopy (Figure 1C, lower panel), axenic amastigotes of *L. mexicana* were fixed and permeabilised as described above before labelling with anti-LmexHASPB (1∶100) and detection with goat-anti-rabbit-FITC secondary antibody (Sigma). Fluorescent parasites were viewed using a Nikon Microphot FX epifuorescent microscope, images captured with a Photometrics CH350 CCD camera and data analysed via IPLab Spectrum software (Scanalytics). Intramacrophage *L. mexicana* amastigote infections were carried out as described above for *L. braziliensis,* except that macrophages were grown on glass coverslips. Infected macrophages were fixed and permeabilised as described above. HASPB localisation (Figure 1C, upper panel) was determined using anti-Lmex HASPB, with detection by AlexaFluor-488-conjugated goat anti-rabbit IgG (1∶250; Invitrogen).

For analysis by flow cytometry, parasites were labelled live as described above. Samples were analysed on a Dako CyAn ADP and data evaluated by Summit 4.3 Software.

### Clinical samples

Sera samples taken from 6 patients, from whom *L. (V.) braziliensis* parasites were also isolated, were kindly provided by the Leishmaniasis Immunobiology Laboratory, Institute of Tropical Pathology and Public Health, Goiás Federal University (Leishbank - IPTSP/UFG/GO; see Table 1). The blood samples were collected as part of the initial diagnostic procedure, at the time of first clinical evaluation and prior to treatment.

## Results

### The *L. (L.) mexicana* HASPB genes are expressed predominantly in intracellular amastigotes

The LmcDNA16 locus, encoding HASP and SHERP proteins, is conserved in all New and Old World *L. (Leishmania)* species analysed including *L. (L.) major, L. (L.) donovani, L. (L.) infantum, L. (L.) mexicana* and *L. (L.) amazonensis* [3], [8], [9], [10], [11], [38]. In the two New World *L. (Leishmania)* species, *L. (L.) mexicana* and *L. (L.) amazonensis,* the LmcDNA16 locus on chromosome 23 contains several HASP genes [39]. However, unlike in *L. (L.) major* in which HASPB sequences are expressed highly in both metacyclics and amastigotes, HASPB expression occurs predominantly in amastigotes, both at the RNA and protein level, in species of the *L. (L.) mexicana* complex (Figure 1). RNA blotting with the NREP probe overlapping the central repetitive region of the predicted HASPB open reading frame (ORF) detects a single 2 Kb transcript in both *L. (L.) mexicana* and *L. (L.) amazonensis* that is ∼10-fold more abundant in axenic amastigotes than in metacyclic promastigotes and barely detectable in procyclic parasites (Figure 1A). This expression pattern correlates with that observed at the protein level, using an antibody raised against recombinant protein expressed from the central repetitive region of the *L. (L.) mexicana* ORF to detect wild type proteins in lysates of the different parasite stages in both species (Figure 1B). A single HASPB protein of ∼35 kDa (*L. (L.) mexicana*) and ∼29 kDa (*L. (L.) amazonensis*) is detected by immuno-blotting in axenic amastigotes only. As observed with *L. (L.) major* HASPB, these proteins run aberrantly when separated by SDS-PAGE [10]; the molecular masses deduced from the gene sequences are 18.6 kDa and 14.9 kDa respectively. Fluorescence microscopy using the same antibody shows localisation of the HASPB protein in a punctate pattern at the plasma membrane of both axenic and intra-macrophage parasites in *L. (L.) mexicana* (Figure 1C) and also, in *L. (L.) amazonensis* (data not shown).

These data confirm that the HASPBs of the *L. (L.) mexicana* complex are differentially regulated during the parasite life cycle, as in *L. (L.) major,* but unexpectedly, expressed predominantly in the macrophage-dwelling amastigotes.

### Replacement of the LmcDNA16 locus in *L. (Viannia)* species

**Figure 2:**
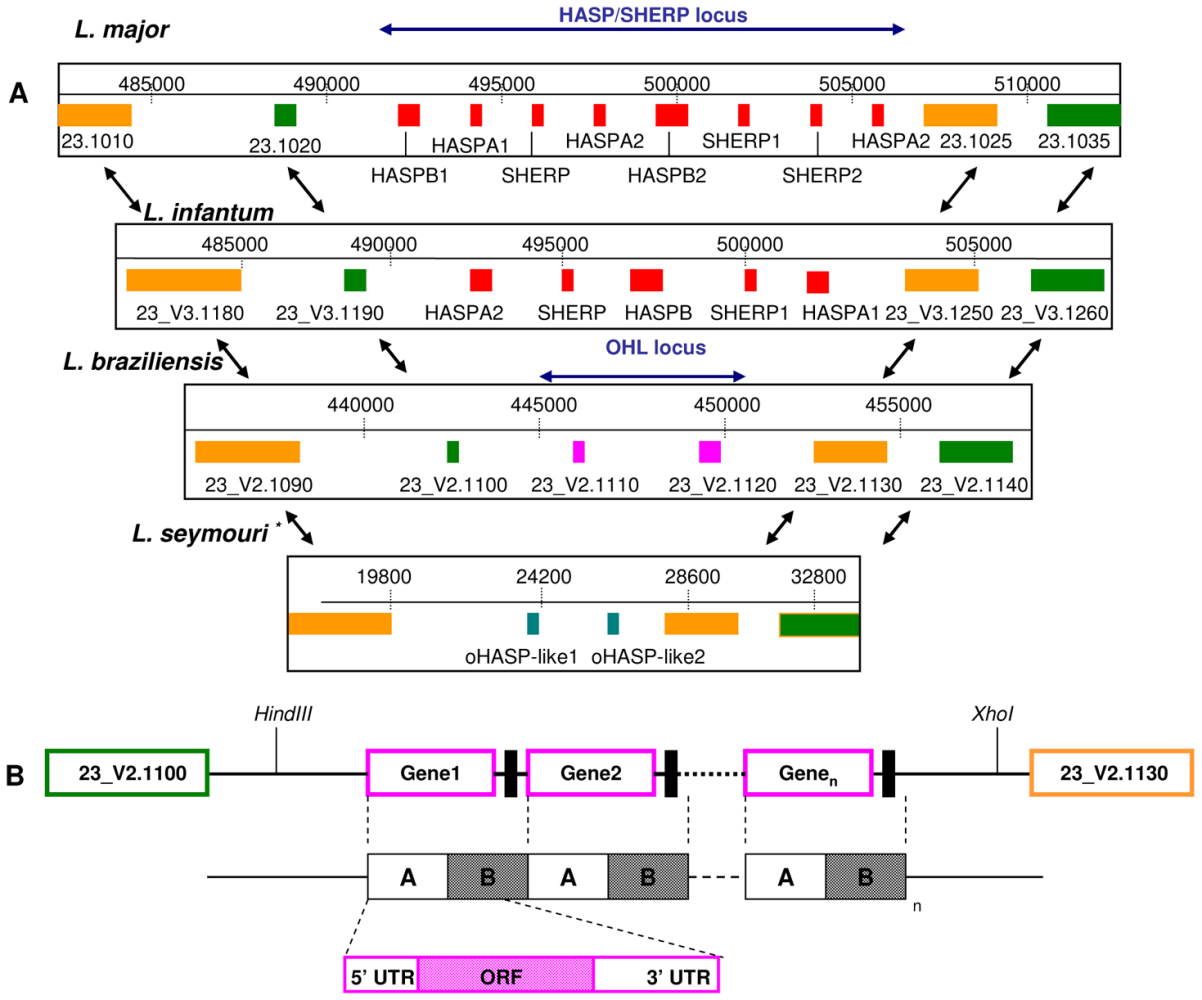
Alignment of the HASP/SHERP loci and related regions in *Leishmania* species. A. Alignments of chromosome 23 from three *Leishmania* species (*L. (L.) major*, *L. (L.) infantum*, *L. (V.) braziliensis*) and the syntenic region of a partial *Leptomonas seymouri* assembly, showing the HASP/SHERP (or LmcDNA16) loci of *L. (L.) major* and *L. (L.) infantum* flanked by conserved syntenic regions that extend in excess of 50 kb in each direction. The closest flanking orthologous genes are linked by angled arrows. Gene colours indicate their current annotation status: red, experimentally characterised; orange, orthologous genes present in other genera; green, orthologous genes present only within the *Leishmania* genus; pink, genes unique to single *Leishmania* species. The OHL locus of *L. (V.) braziliensis* is also shown located in the same position as the HASP/SHERP locus in the Old World species with the same flanking orthologous genes. The syntenic region from the draft sequence of *L. seymouri* reveals two genes (blue) that show similarity to the unique genes in *L. (V.) braziliensis*. * note that the draft assembly for *L. seymouri* has no gene IDs assigned and the position numbers do not reflect the actual position of the locus on the chromosome. B. Representative map of the *L. (V.) braziliensis* OHL locus (deduced from this study; not to scale). Restriction sites used for blotting analysis and probe hybridisation sites (vertical black bars within intergenic regions) are shown. The collapsed repeat identified within this locus contains two distinct regions (A, 1.2 k and B, 0.8 kb) with the conserved unique gene (Lb1120) overlapping both fragments as shown . The copy number of the AB motif has not been accurately determined but is estimated to occupy no less than 15 Kb of chromosomal DNA (estimated from Southern Blot data, Figure S1).

Although conserved in *L. (Leishmania)* species, the LmcDNA16 locus was reported as missing in *L. (V.) braziliensis*, one of the few chromosomal regions showing significant divergence between *Leishmania* species sequenced to date [3]. Instead, a non-syntenic region of ∼7Kb (named here the OHL locus) is positioned at the same chromosomal location in *L. (V.) braziliensis*, as determined by examination of the LmcDNA16 locus flanking regions that contain genes that are conserved in all sequenced *L. (Leishmania)* species (Figure 2A). In addition, partial genome sequencing of *Leptomonas seymouri*, a related insect parasite, has identified a similar variable region between the same conserved flanking genes which is of reduced size and contains two ORFs (Figure 2A).

To confirm the content of the *Leishmania* loci, PCR amplification was used to probe *L. (Viannia)* and *L. (Leishmania)* species for HASPB and SHERP sequences, as well as for the two new ORFs identified in the OHL region of *L. (V.) braziliensis* (LbrM23V2.1110 and LbrM23V2.1120). This analysis confirmed the presence of conserved HASPB and SHERP genes in all analysed *L. (Leishmania)* species and their absence in *L. (Viannia)* species (data not shown). Similarly, the newly identified ORFs were only detected in the *L. (Viannia)* species although notably, the sizes of the bands observed were variable in both number and size (data not shown). Previous studies have shown that the genes within the LmcDNA16 locus exhibit both inter- and intra-species variation in size and content [11], [15]. Similar variation in the size of the OHL region was demonstrated by hybridisation analysis of genomic DNAs from *L. (V.) braziliensis, L. (V.) peruviana* and *L. (V.) guyanensis* (Figure S1). Southern blots of *Hin*DIII/*Xho*I-digested DNA (utilising restriction sites flanking the *L. (V.) braziliensis* OHL region) probed with a specific intergenic fragment (located between LbrM23V2.1110 and LbrM23V2.1120; see Figure 2B) identified single bands of different sizes larger than 12 Kb in *L. (V.) braziliensis*, *L. (V.) guyanensis* and *L. (V.) peruviana* DNA. These fragments were all considerably larger than the ∼7 Kb predicted to span the break in chromosomal synteny derived from *L. (V.) braziliensis* genome analysis (Figure 2A).

**Figure 3:**
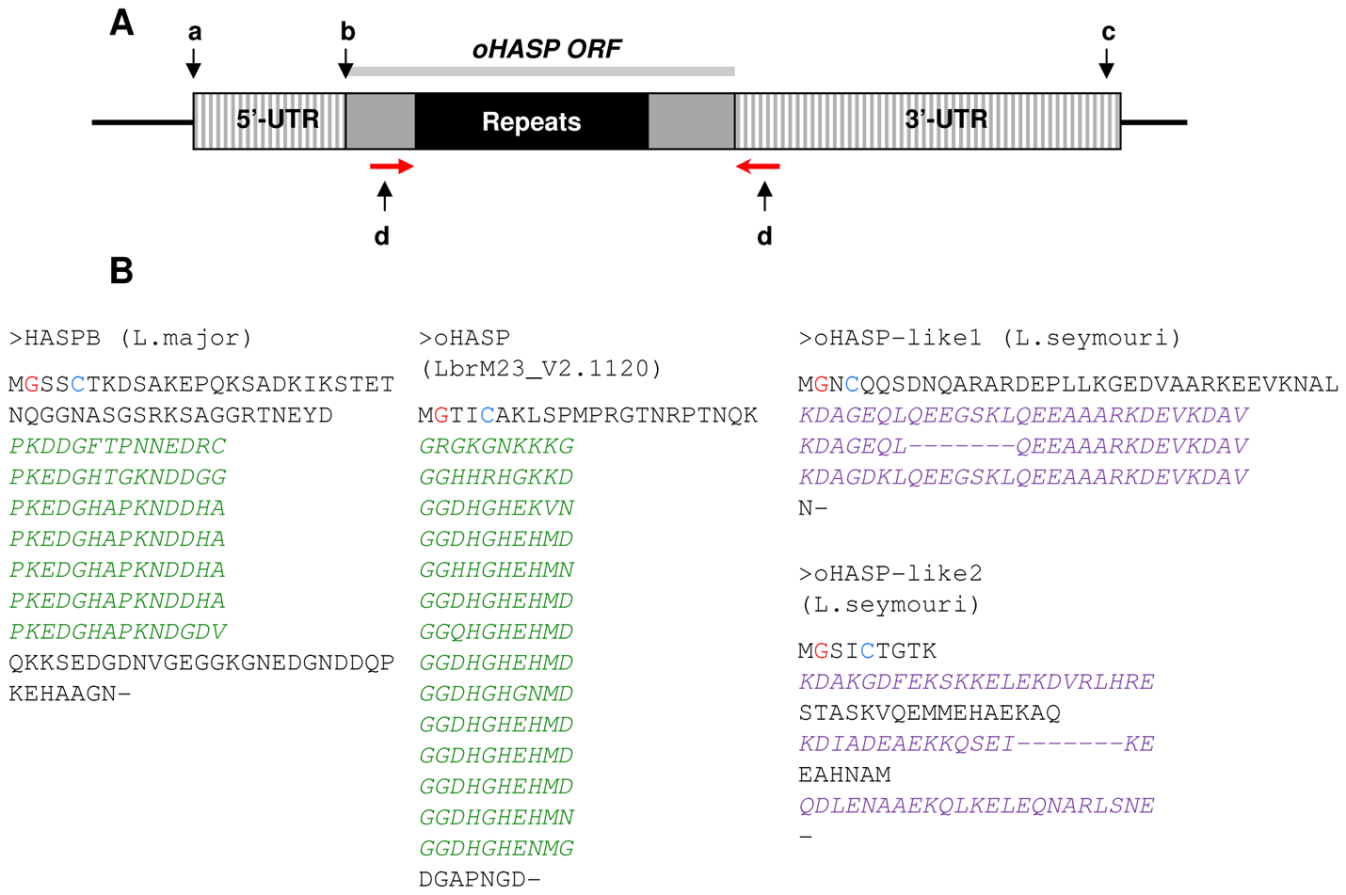
Structure of an oHASP gene and ORF sequences of related proteins. A. One copy of a *L. (V.) braziliensis* oHASP gene is shown (not to scale). The 5′-UTR is defined by a *trans*-splicing acceptor site (a) and translation initiation site (b), identified using consensus sequences derived by [42]. The putative polyadenylation site (c) was predicted by PREDATERM [43]. Within the oHASP ORF (grey bar), the central black domain represents the amino acid repeats, which comprise >60% of the ORF. Primers designed to amplify the repeat region are indicated by the red arrows (d) and are shown in Table S1. B. Sequences of HASPB and oHASP ORFs from *L. (L.) major*, *L. (V.) braziliensis* and *L. seymouri.* The repeats are italicised in each protein; those highlighted in green show inter- and intra-species variation in the number of repeat units present within these multicopy ORFs; the putative sites for *N* –myristoylation and palmitoylation, determined experimentally in *L. (L.) major* HASPB [13], are indicated (red and blue respectively).

Additional bioinformatics analysis revealed a sequence mis-assembly derived from a ∼3.2 kb collapsed repeat sequence within the OHL locus. Collapsed repeats of this type frequently arise during automated genome assembly when sequence reads originating from distinct repeat copies are incorrectly joined to generate a single unit. They are identified as genomic regions with significantly increased read depth. The collapsed repeat identified here contains conserved ∼1.2 kb sequences (A) flanked by ∼0.8 kb sequences (B) forming an ABAB motif, as shown in Figure 2B. Each A sequence contains a putative ORF containing multiple iterations of conserved 30 nt sequences that code for a large amino acid repeat domain (see Figure 3). The number of repetitive 30 nt sequences varies between the two ORFs identified in GeneDB (http://www.genedb.org) as LbrM23V2.1110 and LbrM23V2.1120, with 4 and 14 iterations respectively. It is important to note however that these two ORFs differ only in the number of repeat units present.

Examination of the individual sequence reads that map to the collapsed repeat region reveal the presence of another ORF variant (containing 12 iterations of the repeat motif). While only three variant ORFs were detected in this analysis, the increased read depth within the OHL region suggests that multiple copies of each motif could be present and that the structure of this repeat region consists of a tandemly repeated ABAB pattern, with sequence diversity within the iterated sequences, spanning more than 12 Kb of genomic DNA. Further analysis to more precisely define the size and composition of the OHL locus is in progress.

### Characterisation of the putative ORFs within the *L. braziliensis* OHL region

From the analysis above, the two ORFs identified within the OHL region (LbrM23V2.1110 and LbrM23V2.1120) represent only part of the coding capacity of this domain; there are several more related genes that are not mapped within the OHL locus representation shown in Figure 2. Focusing on the sequence of the single LbrM23V2.1120 ORF, features characteristic of *Leishmania* genes were identified: a translation initiation site (Figure 3Ab) with an upstream AG splice acceptor site flanked by a conserved consensus sequence motif (^−12^cCNcccNcNCAGNaN(C/T)N^+5^; Figure 3Aa) preceded by a long polypyrimidine tract. CLUSTALW alignments of all putative ORFs identified in this locus, together with their flanking regions, revealed strong conservation of the 5′- and 3′-UTRs and putative conserved splice acceptor sites ∼230 nt upstream of the translation initiation site `(data not shown). While 3′ polyadenylation (poly A) sites show significant variation between characterised *Leishmania* genes and cannot usually be identified by simple sequence consensus motifs, use of the PREDATERM program here (which predicts poly A sites based on local nucleotide composition) facilitated identification of putative poly A sites within the flanking B sequences of the oHASP genes (as positioned in Figure 3Ac). This information suggested that the 3′-UTRs of these genes are extensive, in common with other *Leishmania* genes. While these predicted RNA processing sites require experimental verification, their positions confirm that the OHL genes span the A and B sequences in Figure 2B, with the repeats arranged in an AB, AB reiterating pattern for RNA expression.

Comparative analysis of the putative proteins encoded by the OHL ORFs revealed significant conservation although, as described above, variation was observed in the composition and number of iterations of the 30 nt repeats that code for hydrophilic 10 amino acid repeats (Figures 3B). Of particular interest is the presence of conserved N-terminal residues, including a 2^nd^ position glycine and a 5^th^ position cysteine, confirmed as potential sites for *N*-myristoylation and palmitoylation using the NMT- The MYR Predictor and CSS-PALM predictive tools [27]–[28]. By contrast, screening for potential prenylation sites (by PrePS), GPI-modification sites (by big-PI Predictor) or GPI-anchor signal sequences (by GPI-SOM ) returned no positive predictions. Overall, these data indicate that the AB sequence repeats embedded within the OHL locus have the necessary sequence components for identification as functional genes coding for proteins that contain large internal hydrophilic repeat domains and may be modified both co- and post-translationally by N-myristoylation and palmitoylation. The OHL ORFs, therefore, have very similar characteristics to the *L. (Leishmania)* HASPB proteins, features evident in the comparisons and alignments presented in Figure 3 and Figure S2.

A similar analysis of the two *L. seymouri* ORFs reveals that both contain large hydrophilic amino acid repeat domains that are larger than those observed in the HASPs and oHASPs and also more degenerate (Figure 3B). Predicted sites for *N-*myristoylation and palmitoylation are also found in these deduced protein sequences (Figure 3B).

### Expression and localisation of the OHL gene products

**Figure 4:**
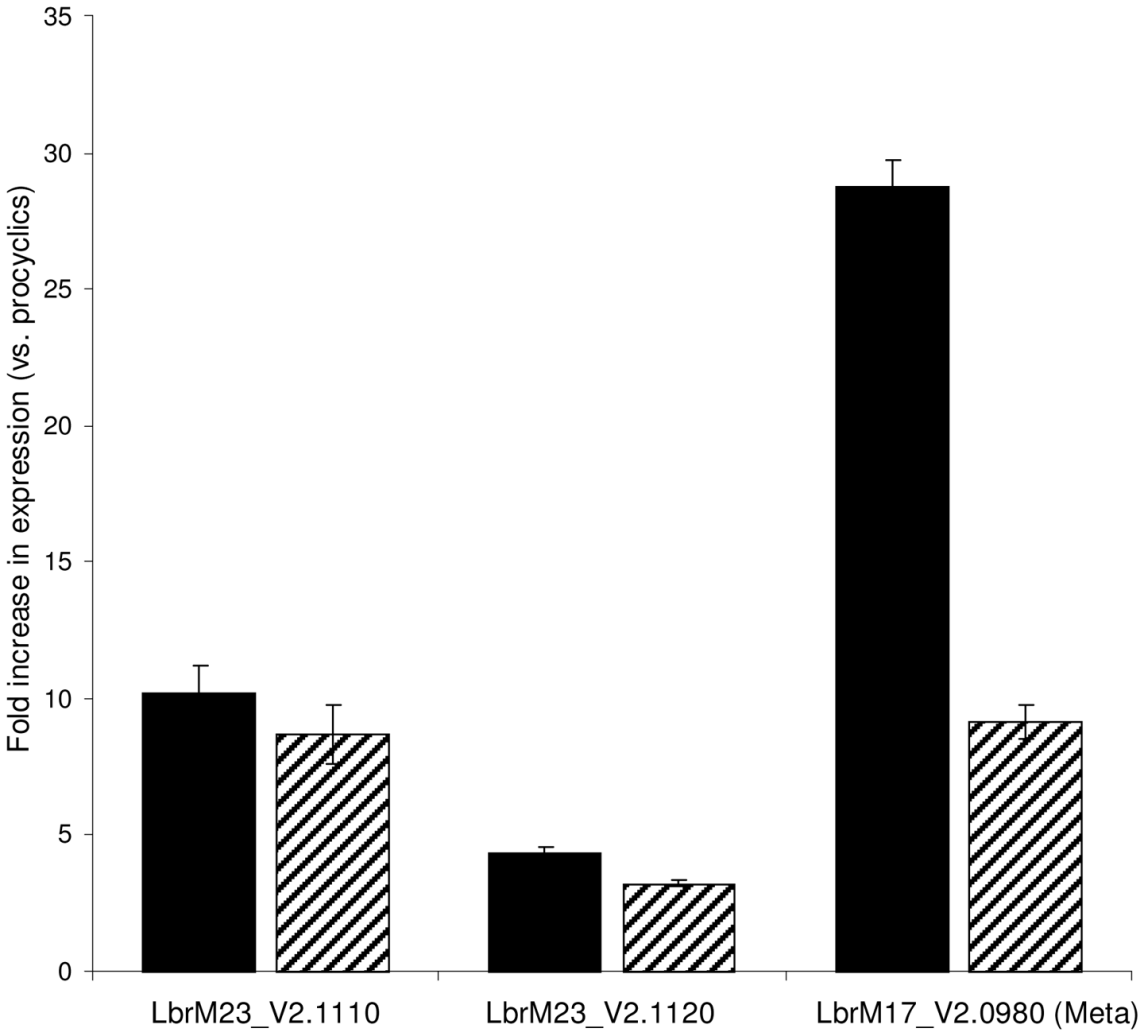
Expression profiling of *L. (V.) braziliensis* oHASP genes. Quantitative analysis of RNA expression in procyclic, metacyclic and amastigote stages of *L. (V.) braziliensis* was carried out by RT-qPCR as described [33] with results displayed as ratios of fold increase in expression in metacyclics (black bars) and amastigotes (hatched bars) relative to procyclic parasites. Error bars represent standard errors of the mean. In this analysis, γ-glutamyl cysteine synthetase (LbrM18_V2.1700) was used as a constitutive control and Meta1 (LbrM17_v2.0980) as a stage-specific control for metacyclic parasites [36].

To investigate RNA expression from the two characterised OHL genes (LbrM23_V2.1110 and LbrM23_V2.1120), RT-qPCR was used for quantitative analysis of transcript levels in macrophage-derived amastigotes and axenic procyclic and metacyclics of *L. (V.) braziliensis*. The results were normalised using the experimentally-characterised γ-glutamyl cysteine synthetase (LbrM18_V2.1700) as a constitutive control and Meta1 (LbrM17_V2.0980) as a marker for metacyclic expression [36]. Data were analysed using the Pfaffl method [33] and showed that the transcript abundances from both genes are increased 5 – 10 fold in the infective metacyclic and amastigote stages relative to the procyclic stages of the parasite (Figure 4). As expected, expression of the Meta1 gene was highly up-regulated in metacyclics versus amastigotes, as previously reported [36]. These observations show strong correlation with the stage-regulated transcript abundances of the HASPB genes in *L. (L.) major* [8] but less so with the *L. (L.) mexicana* species, that show predominant RNA expression in amastigotes, by RNA blotting and hybridisation analysis (Figure 1).

**Figure 5:**
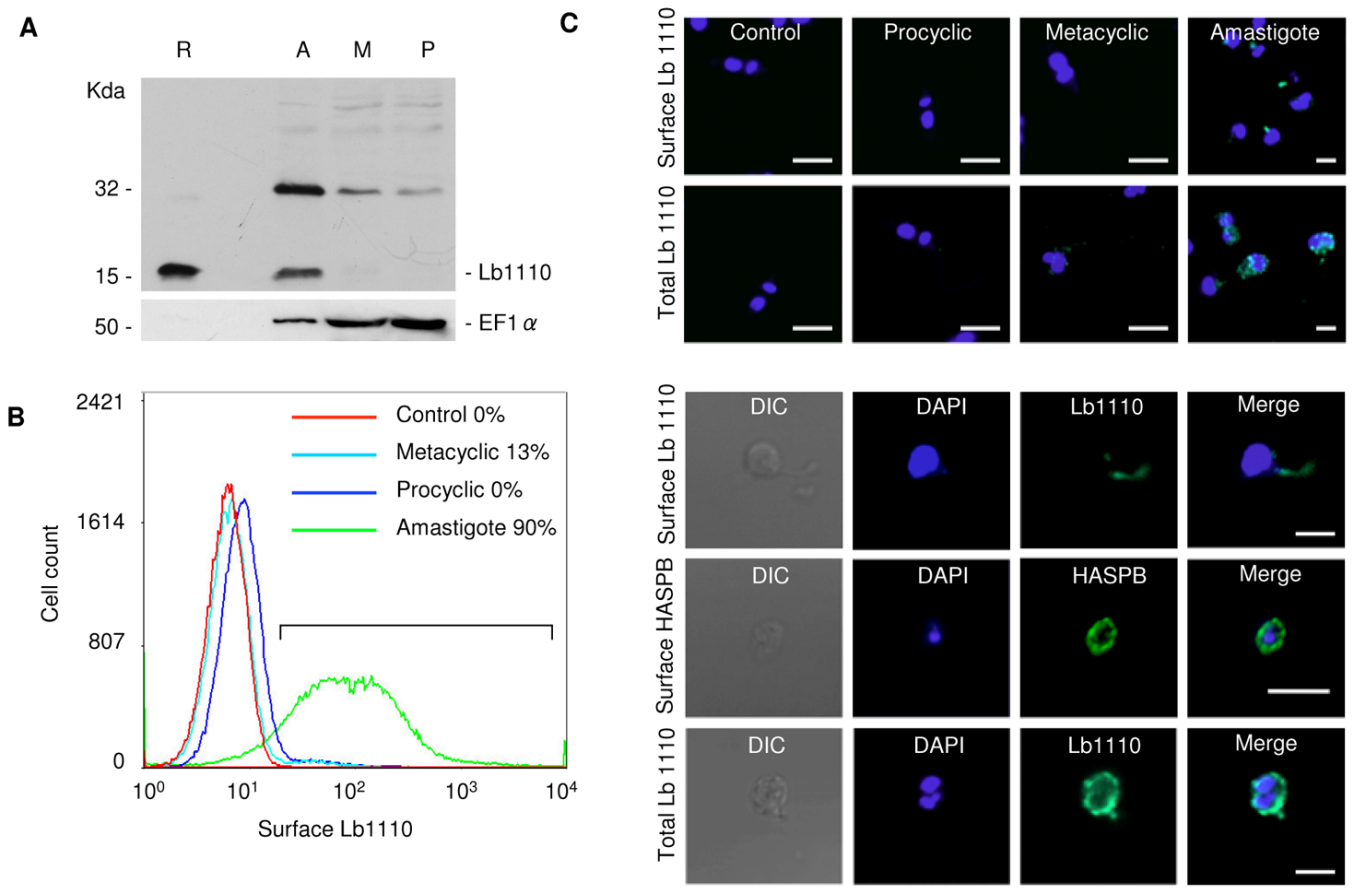
Expression and localisation of Lb1110 protein in *L. (V.) braziliensis.* A. Immunoblotting analysis of Lb1110 expression in procyclics (P), metacyclics (M) and amastigotes (A) of *L. (V.) braziliensis* (strain M2904-75). Recombinant Lb1110 (R), migrating as a 15 kDa protein on SDS-PAGE, was used to generate anti-Lb1110, the antiserum used to probe the blot shown, loaded with total protein lysates from the different parasite stages. Anti-EF1α was used as a constitutive control for protein loading on the re-probed blot below. B. Analysis of Lb1110 expression by flow cytometry in live parasites using the antibody described in A. Surface-exposed Lb1110 was detected by live primary antibody labelling prior to fixation and detection with AlexaFluor 488-conjugated goat anti-rabbit IgG. Total Lb1110 was detected by antibody-labelling post-fixation. Prior to live cell staining, the amine-reactive fluorophore sulfo-succinimidyl-7-amino-4-methylcoumarin-3-acetic acid (Sulfo-NHS-AMCA) was used to confirm cell viability; dead cells stained with this reagent emit a strong blue fluorescence and can be omitted from further analyses. The experiment shown was one of two conducted, both of which showed similar % cell counts. Control, no primary antibody. C. Use of confocal microscopy to detect either total or surface-exposed Lb1110 in *L. (V.) braziliensis* stages (top panel); control, no primary antibody used. Amastigotes only of *L. (V.) braziliensis* and *L. (L.) mexicana* (bottom panel) are shown as DIC (differential interference contrast) images and following staining with DAPI, anti-Lb1110 or anti-HASPB, either pre- or post-fixation for surface or total protein distribution. Scale bars, 5 µm.

To further probe gene expression at the protein level, the OHL gene encoding the smallest number of repeats (LbrM23_V2.1110 or Lb1110) was chosen for detailed analysis. Using an affinity-purified antibody raised against recombinant Lb1110 expressed in *E. coli* (see Materials and Methods), several approaches were used to investigate Lb1110 expression in the different *L. (V.) braziliensis* life cycle stages (Figure 5). Firstly, immunoblotting detected strong reactivity with the 15 kDa recombinant protein and recognised a protein of the same size only in amastigotes of *L. (V.) braziliensis* (Figure 5A). In view of the RNA expression analysis above, this observation suggests that expression of the Lb1110 gene may be regulated translationally, unlike the *L. (L.) mexicana* homologue described in Figure 1. A second protein, migrating at ∼2x the observed molecular mass of recombinant Lb1110, was also detected by anti-Lb1110 in amastigote lysates although this molecule was also detectable at low levels in metacyclics and procyclics (when compared to the differential loading indicated by detection of the constitutive marker, EF1α). It is likely that this protein is an additional oHASP containing a more extended repeat domain that is recognised by anti-Lb1110. Other OHL genes may also be expressed as proteins that are of lower abundance and therefore not readily detectable by immunoblotting.

Flow cytometry was used as a second method to quantify Lb1110 protein surface expression in procyclic, metacyclic and amastigote *L. (V.) braziliensis*. In these experiments, surface-exposed Lb1110 was detected by live primary antibody labelling prior to fixation and detection with AlexaFluor 488-conjugated goat anti-rabbit IgG (see Materials and Methods). Prior to live cell staining, the amine-reactive fluorophore sulfo-succinimidyl-7-amino-4-methylcoumarin-3-acetic acid (Sulfo-NHS-AMCA) was used to confirm cell viability; dead cells staining with this reagent emit a strong blue fluorescence and could be omitted from further analyses. As shown in Figure 5B, live cell staining with anti-Lb1110 was not detectable in control (no primary antibody used) or procyclic parasite populations. Conversely, 13% of parasites in a metacyclic population stained with anti-Lb1110 while 90% of amastigotes were positive with this antibody. These results confirm the stage-specificity of Lb1110 expression and demonstrate its surface exposure on the majority of amastigotes.

As a third approach to determining the localisation of the Lb1110 protein, expression was visualised in either live or permeabilised and fixed *L. (V.) braziliensis* by indirect immunofluorescence and confocal microscopy (Figure 5C). Antibody labelling was carried out either pre- or post-fixation at 20°C, in order to compare antigen localisation at the surface membrane with that detected both externally and internally within the parasite. DAPI staining of the parasite nucleus and kinetoplast was used as a counter-stain in these experiments. As shown in the upper panel of Figure 5C, anti-Lb1110 staining is specific to *L. (V.) braziliensis* amastigotes and, in live antibody labelled cells, Lb1110 localises to a site close to the protrusion of the rudimentary flagellum, which could be indicative of antibody capping of the surface exposed protein. In permeabilised cells (labelled Total Lb1110), by comparison, staining is evident in a punctate pattern indicative of plasma membrane and flagellar localisation on both faces of the membrane bi-layer. In the lower panel of Figure 5C, a single *L. (V.) braziliensis* amastigote is shown at higher magnification, clearly demonstrating the plasma membrane localisation following permeabilisation but surface localisation to the rudimentary flagellum in the non-permeabilised *L. (V.) braziliensis* cell. In contrast, the live labelling pattern on the *L. (L.) mexicana a*mastigote in the same figure (‘Surface HASPB’) is very similar to the total labelling pattern on the fixed *L. (V.) braziliensis* amastigote (and to the fixed labelling seen in Figure 1C), suggesting that antibody capping is minimal on live *L. (L.) mexicana* under the labelling conditions used. Overall, these data suggest that the surface distribution of Lb1110 to the amastigote flagellum is not an artefact of antibody capping in these live cells.

**Figure 6:**
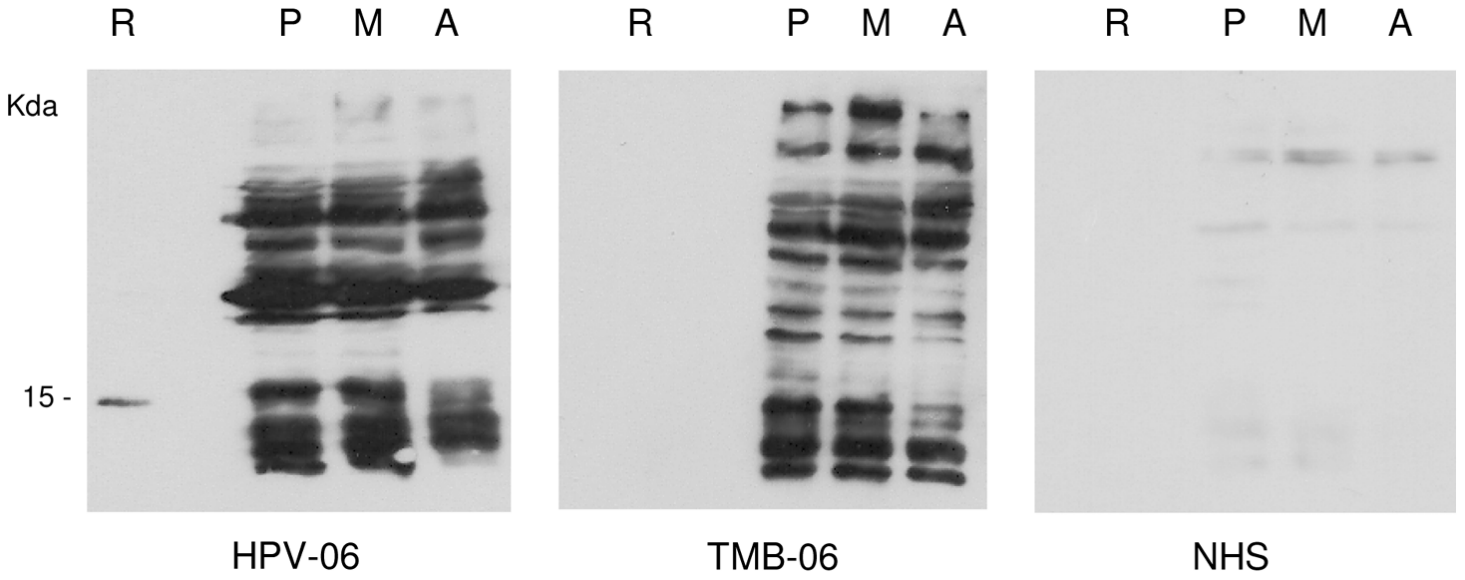
Immune recognition of recombinant Lb1110 and total parasite proteins by human sera. Samples of the same protein extracts analysed in Figure 5A were separated by SDS-PAGE, blotted and probed with human antiserum (at 1∶300 – 1∶500 dilution) collected from patients that were the source of two of the clinical isolates listed in Figure 7 (HPV-06, TMB-06). NHS, normal human serum.

Given the amastigote-dominant expression of Lb1110 and its surface exposure on live parasites, in a pattern similar to that observed for HASPB expression in *L. (L.) major* metacyclic parasites [40], we next investigated whether this protein is recognised by human immune serum collected from patients infected with *L. (V.) braziliensis*. Six serum samples derived from infections with the *L. (V.) braziliensis* clinical isolates listed in Table 1 were used to probe blots of separated parasite proteins from different stages, together with recombinant protein (as used in Figure 5A). Two examples of these immunoblots, representative of the patterns observed, are shown in Figure 6, probed with serum taken from HPV-06 and TMB-06 infections (using the same serum dilution and length of chemical exposure for all blots). Recognition of a broad size range of proteins in total parasite extracts was evident in all stages with each antiserum, while normal human serum detected few proteins above background levels and did not recognise recombinant Lb1110. Interestingly, the recombinant protein was strongly detected by HPV-06 but not by TMB-06. Overall, these data confirm the antigenicity of Lb1110 and, as with the central repetitive domain of *L. (L.) major* HASPB, it can be predicted that the Lb1110 repeats may provide dominant epitopes for antibody recognition. It is also evident that not all antisera taken from infected patients recognise Lb1110, suggesting that this antigen could be unstable or variant *in vivo*. To investigate this further, oHASP gene repeats were analysed in a number of *L. (V.) braziliensis* clinical isolates (provided as genomic DNAs from Leishbank - IPTSP/UFG/GO and listed in Table 1).

### Sequencing of the variable repeat domains within the oHASPs

Variation in the number of repeat iterations present in each of the 2 OHL ORFs described above (LbrM23V2.1110 and LbrM23V2.1120), coupled with the large size of the non-syntenic region, raised the possibility that further ORFs with distinct repeat regions might be present in this region, as discussed earlier. To verify this prediction, genomic DNA from the *L. (V.) braziliensis* genome strain (MHOM/BR/75/M2904) was subjected to PCR with primers designed to amplify the repetitive domain in the oHASPs (Figure 3Ad, Table S1). The PCR products were sub-cloned into the pGEM-T-easy vector, 10 clones of each selected and their insertions sequenced. The repeat domain structure was then determined for each clone and each unique sequence translated and aligned using CLUSTALW. Three unique sequences were identified (comprising 9, 12 and 14 repeat units) and aligned (Figure S3) revealing variations in the number and sequences of the repeat domains of the oHASP ORFs in a single strain.

**Figure 7:**
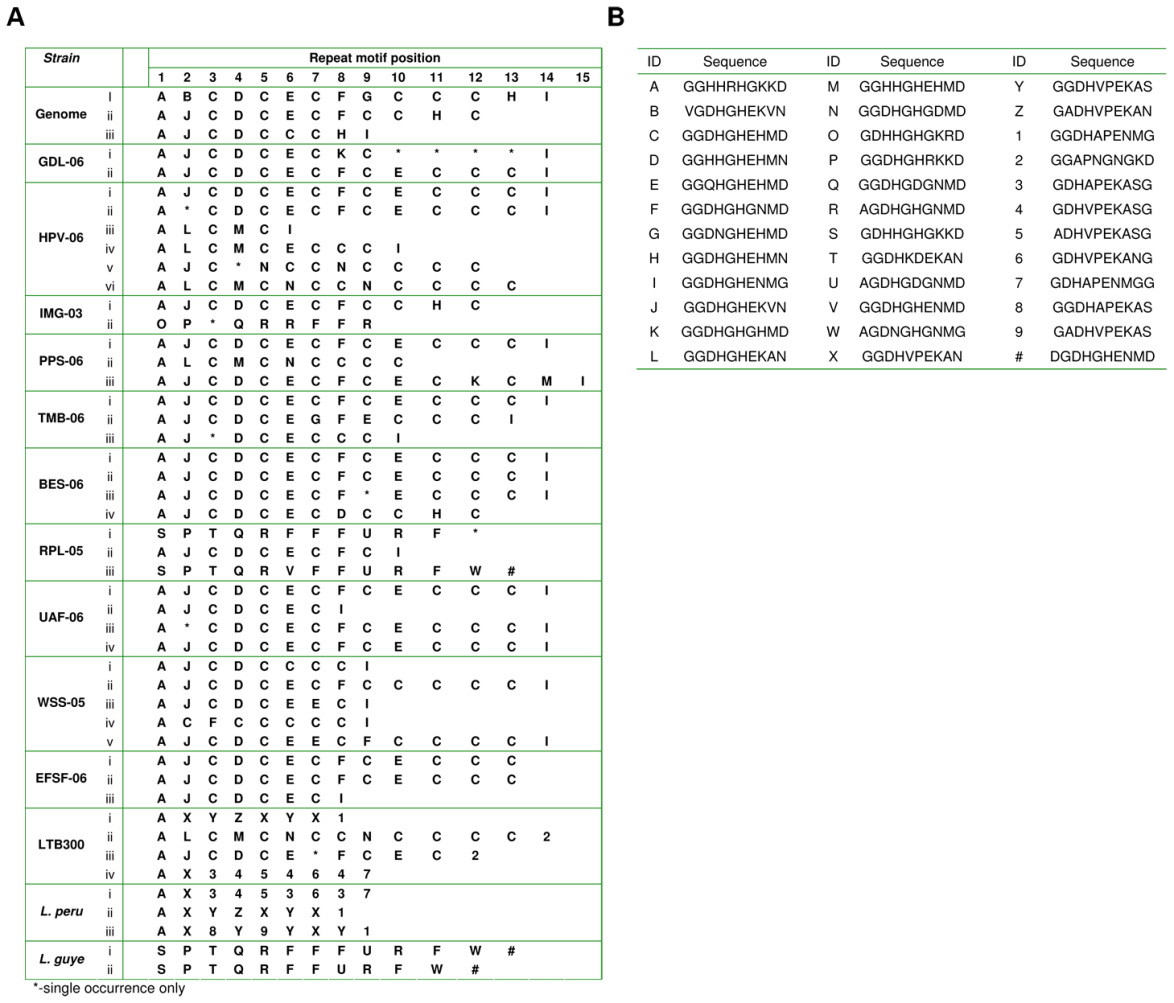
Distribution of repeat units within repeat domains of OHL variants from *L. (Viannia)* species. PCR amplification, cloning and sequencing were used to analysis the OHL repeat domains in strains of *L. braziliensis*, *L. peruviana* and *L. guyanensis* (described in Table 1). Each unique OHL sequence identified per strain (i - vi) was analysed and the position (within the repeat domain, shown in A) and composition of each individual 10-amino acid repeat unit (lettered A-Z, numbered 1-9 plus #) recorded in B. Sequences marked as single occurrence only (*) contain several low quality reads; a reliable sequence could not be definitively determined.

The variations in size and composition of the repeat domains of the *L. (V.) braziliensis* genome strain oHASPs, as described above, are similar to those reported in the repetitive domains of *L. (L.) major* and *L. (L.) donovani* HASPBs [11], [15] and typical of the observed inter- and intra- species variation in this protein family in the *L. (Leishmania)* subgenus. To determine the extent of similar variation in the *L. (Viannia)* oHASPs, a total of 11 isolates of *L. (V.) braziliensis* (Table 1) and single strains of *L. (V.) peruviana* and *L. (V.) guyanensis* were analysed, using the same approach as above, cloning and sequencing multiple clones of each strain. These data are presented in Figure 7, which includes the organisation of translated repeat domains in up to 4 independent clones from each PCR amplification (A) and the composition of each repeat unit analysed (B).

The number of distinct repeat domains identified in each strain of *L. (V.) braziliensis* varied from 2–6 per strain. The average repeat domain comprised 14 iterations with 6 being the lowest observed and 15 the highest (Figure 7). While the composition of the repeat domains varied both between and within strains, it is interesting to note that the most prevalent motif within the repeat region across all strains (excepting LTB300 and RPL-05) is GGDHGHEHMD (Figure 7B, sequence G). Also of note, the repeat domains in the LTB300 strain are very similar to those observed in *L. (V.) peruviana* strain LpLCA08-90. Similarly, the RPL-05 strain shares greater conservation with the *L. (V.) guyanensis* strain LgM4147-75 repeat domains than with the *L. (V.) braziliensis* genome strain (M2904-75). In these cases, the most prevalent repeat unit appears to be GGDHVPEKAN (Figure 7B, sequence X) and GGDHGHGNMD (Figure 7B, sequence F) respectively. Intriguingly, the overall structure of the repeat domains is well conserved between the representative sequences from each strain with the individual motifs occupying very specific positions (Figure 7). The functional significance of this conservation has not yet been investigated further.

In comparison to *L. (V.) braziliensis*, considerable variation in the repeat domains was observed for both *L. (V.) peruviana* and *L. (V.) guyanensis* (although only a single isolate of each species was investigated). Analyses of these sequences show the level of conservation at the amino acid level between *L. (V.) braziliensis* and *L. (V.) guyanensis* to be ∼76%, *L. (V.) braziliensis* and *L. (V.) peruviana* ∼62% and *L. (V.) guyanensis* and *L. (V.) peruviana* ∼57%, suggesting that the *L. (V.) peruviana* repeat domain is the most divergent in content (Figure 7). Moreover the average size of the oHASP repeat domain is significantly less in *L. (V.) peruviana* (Figure 7). These species-specific variations in the size and content of the repeat domains are similar to those observed in the HASPB sequences in *L. (Leishmania)* species.

## Discussion

The LmcDNA16 locus, identified on chromosome 23 in all *L. (Leishmania)* species examined to date, contains two unusual and apparently unrelated gene families (encoding the HASPs and SHERPs), both of which are preferentially expressed during infective stages of the parasite life cycle. Ongoing functional characterisation using transgenic parasite lines lacking this locus has revealed an essential role for members of these gene families in facilitating differentiation of *L. (L.) major* parasites in the sandfly vector, *Phlebotomus papatasi* [41]. These observations suggest that the HASP and/or SHERP proteins are also likely to be essential for parasite transmission from vector to host in *L. (L.) major*. The absence of the HASP/SHERP locus from the *L. (V.) braziliensis* genome assembly, and the identification in this study of the distinct, if related, OHL region encoding proteins that also contain amino acid repeats and localise predominantly to the amastigote (but not metacyclic) plasma membrane, raises questions regarding the role of these parasite proteins in transmission from vector to host in *L. (Viannia)* species.

The data generated in this study demonstrate that the OHL and LmcDNA16 loci are subgenus specific (found in *L. (Viannia)* and *L. (Leishmania)* respectively), yet probably arose from a common ancestor, as suggested by analysis of the syntenic region in the monogenetic *L. seymouri.* Interestingly, in all *Leishmania* species examined so far, this region of chromosome 23 encodes gene families with similar features. These include (a) the presence of large hydrophilic amino acid repeat domains within proteins that are potentially N-terminally acylated; and (b) localisation and exposure of at least one of the encoded proteins at the plasma membrane during infective stages of the parasite life cycle. The similarity in expression patterns and localisation of the HASP and oHASP proteins supports the proposal that the encoding genes are orthologous.

The HASPBs have been previously shown to be recognition targets for host immune responses [16], [17], [18], [19], [20], possibly due to their high charge and the presence of extended hydrophilic amino acid repeat domains. Intriguingly, the variations observed in the size and composition of the oHASP repeats, both between *L. (Viannia) species* and within *L. (V.) braziliensis* strains, are similar to those observed in the HASPBs. These data support the proposal that the oHASP and HASPB proteins may have conserved functions, although the role of the repeat domains in both proteins is still unclear. While amino acid repeats are frequently involved in protein-protein contacts and could facilitate key interactions during parasite differentiation in the sand fly, the repeat domains of HASPB (and oHASP) are also expressed and diversified as surface antigens in the host, as reported in *L. major* [15], [16] and in this paper. The detection of Lmex HASPB and Lb1110 predominantly in amastigotes of *L. (L.) mexicana* and *L. (V.) braziliensis* respectively suggests a dominant role for these proteins in the host rather than the vector for these species. Perhaps the significant sequence variation observed between the repeat domains of the oHASP proteins in the clinical isolates of *L. (V.) braziliensis* used here could be a consequence of variable host immune pressure.

### Evolution of the LmcDNA16 loci and LmcDNA16 replacement regions

In addition to the complete genomes of *L. (L.) major, L. (L.) infantum* and *L .braziliensis* [2], [3], sequence data are also currently available for *L. seymouri*, a monogenetic protozoan that parasitizes insects, nematodes and ciliates and is the closest sequenced relative to *Leishmania*. The presence of a syntenically-positioned locus containing ORFs that code for putative N-acylated proteins containing large hydrophilic amino acid repeat domains suggests the presence of this hypermutable locus in the pre-*Leishmania* state. Whether this locus is present in *Crithidia* species remains unknown. Given the comparative simplicity of the locus in *L. seymouri,* the expansion seen in *Leishmania spp.* could be representative of the shift from the monogenetic life cycle of ancestral *Leishmania* to the digenetic life cycle of parasites from the *Leishmania sensu strictu* genus. A key step in this process is the evolution of the parasite-parasitized insect relationship allowing *Leishmania* to use sand flies as their vector. Our recent observation that the LmcDNA16 locus is essential for *L. (L.) major* differentiation in *Phlebotomus papatasi* [41] may be of relevance in this respect.

### Concluding remarks

Recent studies have demonstrated the importance of HASP proteins for *L. major* differentiation in the sand fly vector, while the antigenic properties of these molecules suggest their suitability as targets for vaccine development. Previous comparative genomic analyses of *L. (V.) braziliensis, L. (L.) major and L. (L.) infantum,* however, reported the absence of the HASP/SHERP (or LmcDNA16) locus on chromosome 23 in *L. (V.) braziliensis –* with a smaller non-syntenic locus (the OHL locus) found at that location.

In this paper, we show that the oHASP proteins coded within the OHL locus are orthologues of HASPB, possessing similar expression, localisation and antigenic properties. Of particular interest is the inter- and intra-species variation in the size and composition of the oHASP repeat domains (also observed in HASPBs) which could indicate that host (and/or vector) immune pressure is driving sequence diversification within this locus. Further study is now required to investigate the antigenic properties of the oHASPs, explore their interaction with the host immune system and investigate their utility as diagnostic agents for *L. (L.) Viannia* clinical infections.

## Supporting Information

Figure S1 DNA hybridization analysis indicating the relative size of the OHL locus in *L. Viannia* species. 250 ng of genomic DNA from *L. (V.) peruviana* (Lp), *L. (V.) guyanensis* (Lg) and *L. (V.) braziliensis* (Lb), extracted from strains listed in Table 1 (Lb from strain M290475) were digested with *Xho*I and *HinD*III, size separated through 0.6% agarose and hybridized with a digoxigenin probe targeting a repetitive intergenic region (vertical black bars in Figure 2B). A single hybridizing band was observed in the *L. (V.) guyanensis* and *L. (V.) braziliensis* digests while two weaker bands (black dots) were detected for *L. peruviana*. Molecular markers (M) are shown on the left (Kb).
(0.76 MB TIF)

Figure S2 CLUSTALW alignment of the translated oHASP ORF (Lb1110, containing 14 repeat units) with HASPB sequences from *L. (L.) major*, *L. (L.) infantum* and the translated orthologous ORF identified in *L. seymouri*. *N*-myristoylation and palmitoylation sites are shown highlighted in red and blue respectively; conserved residue(*); conserved substitutions(:); semi-conserved substitution (.)
(0.47 MB TIF)

Figure S3 CLUSTALW alignment of the sequenced OHL ORFs (containing 9, 13 and 14 repeat units) revealing variation in both the sequence and number of repeated motifs in the amino acid repeat domains.
(0.25 MB TIF)

Table S1 Primers used for PCR amplifications in this study.
(0.35 MB TIF)

## References

[1] Murray HW, Berman JD, Davies CR, Saravia NG (2005). Advances in leishmaniasis.. Lancet.

[2] Ivens AC, Peacock CS, Worthey EA, Murphy L, Aggarwal G (2005). The genome of the kinetoplastid parasite, Leishmania major.. Science.

[3] Peacock CS, Seeger K, Harris D, Murphy L, Ruiz JC (2007). Comparative genomic analysis of three Leishmania species that cause diverse human disease.. Nat Genet.

[4] Smith DF, Peacock CS, Cruz AK (2007). Comparative genomics: From genotype to disease phenotype in the leishmaniases.. Int J Parasitol.

[5] Joshi PB, Kelly BL, Kamhawi S, Sacks DL, McMaster WR (2002). Targeted gene deletion in Leishmania major identifies leishmanolysin (GP63) as a virulence factor.. Mol Biochem Parasitol.

[6] Yao C, Donelson JE, Wilson ME (2003). The major surface protease (MSP or GP63) of Leishmania sp. Biosynthesis, regulation of expression, and function.. Mol Biochem Parasitol.

[7] McKean PG, Denny PW, Knuepfer E, Keen JK, Smith DF (2001). Phenotypic changes associated with deletion and overexpression of a stage-regulated gene family in Leishmania.. Cell Microbiol.

[8] Flinn HM, Smith DF (1992). Genomic organisation and expression of a differentially-regulated gene family from Leishmania major.. Nucleic Acids Res.

[9] McKean PG, Delahay R, Pimenta PF, Smith DF (1997). Characterisation of a second protein encoded by the differentially regulated LmcDNA16 gene family of Leishmania major.. Mol Biochem Parasitol.

[10] Flinn HM, Rangarajan D, Smith DF (1994). Expression of a hydrophilic surface protein in infective stages of Leishmania major.. Mol Biochem Parasitol.

[11] Alce TM, Gokool S, McGhie D, Stager S, Smith DF (1999). Expression of hydrophilic surface proteins in infective stages of Leishmania donovani.. Mol Biochem Parasitol.

[12] Rangarajan D, Gokool S, McCrossan MV, Smith DF (1995). The gene B protein localises to the surface of Leishmania major parasites in the absence of metacyclic stage lipophosphoglycan.. J Cell Sci.

[13] Denny PW, Gokool S, Russell DG, Field MC, Smith DF (2000). Acylation-dependent protein export in Leishmania.. J Biol Chem.

[14] Knuepfer E, Stierhof YD, McKean PG, Smith DF (2001). Characterization of a differentially expressed protein that shows an unusual localization to intracellular membranes in Leishmania major.. Biochem J.

[15] McKean PG, Trenholme KR, Rangarajan D, Keen JK, Smith DF (1997). Diversity in repeat-containing surface proteins of Leishmania major.. Mol Biochem Parasitol.

[16] Jensen AT, Gaafar A, Ismail A, Christensen CB, Kemp M (1996). Serodiagnosis of cutaneous leishmaniasis: assessment of an enzyme-linked immunosorbent assay using a peptide sequence from gene B protein.. Am J Trop Med Hyg.

[17] Jensen AT, Gasim S, Moller T, Ismail A, Gaafar A (1999). Serodiagnosis of Leishmania donovani infections: assessment of enzyme-linked immunosorbent assays using recombinant L. donovani gene B protein (GBP) and a peptide sequence of L. donovani GBP.. Trans R Soc Trop Med Hyg.

[18] Stager S, Smith DF, Kaye PM (2000). Immunization with a recombinant stage-regulated surface protein from Leishmania donovani induces protection against visceral leishmaniasis.. J Immunol.

[19] Stager S, Alexander J, Kirby AC, Botto M, Rooijen NV (2003). Natural antibodies and complement are endogenous adjuvants for vaccine-induced CD8(+) T-cell responses.. Nat Med.

[20] Moreno J, Nieto J, Masina S, Canavate C, Cruz I (2007). Immunization with H1, HASPB1 and MML Leishmania proteins in a vaccine trial against experimental canine leishmaniasis.. Vaccine.

[21] Peacock CS, Seeger K, Harris D, Murphy L, Ruiz JC (2007). Comparative genomic analysis of three Leishmania species that cause diverse human disease.. Nat Genet.

[22] El-Sayed NM, Myler PJ, Blandin G, Berriman M, Crabtree J (2005). Comparative genomics of trypanosomatid parasitic protozoa.. Science.

[23] Hertz-Fowler C, Peacock CS, Wood V, Aslett M, Kerhornou A (2004). GeneDB: a resource for prokaryotic and eukaryotic organisms.. Nucleic Acids Res.

[24] Altschul SF, Gish W, Miller W, Myers EW, Lipman DJ (1990). Basic local alignment search tool.. J Mol Biol.

[25] Carver TJ, Rutherford KM, Berriman M, Rajandream MA, Barrell BG (2005). ACT: the Artemis Comparison Tool.. Bioinformatics.

[26] Eisenhaber B, Bork P, Yuan Y, Loffler G, Eisenhaber F (2000). Automated annotation of GPI anchor sites: case study C. elegans.. Trends Biochem Sci.

[27] Maurer-Stroh S, Eisenhaber B, Eisenhaber F (2002). N-terminal N-myristoylation of proteins: prediction of substrate proteins from amino acid sequence.. J Mol Biol.

[28] Zhou F, Xue Y, Yao X, Xu Y (2006). CSS-Palm: palmitoylation site prediction with a clustering and scoring strategy (CSS).. Bioinformatics.

[29] Schonian G, Nasereddin A, Dinse N, Schweynoch C, Schallig HD (2003). PCR diagnosis and characterization of Leishmania in local and imported clinical samples.. Diagn Microbiol Infect Dis.

[30] Uliana SR, Nelson K, Beverley SM, Camargo EP, Floeter-Winter LM (1994). Discrimination amongst Leishmania by polymerase chain reaction and hybridization with small subunit ribosomal DNA derived oligonucleotides.. J Eukaryot Microbiol.

[31] Castilho MS, Pavao F, Oliva G, Ladame S, Willson M (2003). Evidence for the two phosphate binding sites of an analogue of the thioacyl intermediate for the Trypanosoma cruzi glyceraldehyde-3-phosphate dehydrogenase-catalyzed reaction, from its crystal structure.. Biochemistry.

[32] Zauli-Nascimento RC, Miguel DC, Yokoyama-Yasunaka JK, Pereira LI, Pelli de Oliveira MA (2010). In vitro sensitivity of Leishmania (Viannia) braziliensis and Leishmania (Leishmania) amazonensis Brazilian isolates to meglumine antimoniate and amphotericin B.. Trop Med Int Health.

[33] Depledge DP, Evans KJ, Ivens AC, Aziz N, Maroof A (2009). Comparative Expression Profiling of Leishmania: Modulation in Gene Expression between Species and in Different Host Genetic Backgrounds.. PLoS Negl Trop Dis.

[34] Bates PA (1994). Complete developmental cycle of Leishmania mexicana in axenic culture.. Parasitology.

[35] Rozen S, Skaletsky H (2000). Primer3 on the WWW for general users and for biologist programmers.. Methods Mol Biol.

[36] Gamboa D, Van Eys G, Victoir K, Torres K, Adaui V (2007). Putative markers of infective life stages in Leishmania (Viannia) braziliensis.. Parasitology.

[37] Studier FW (2005). Protein production by auto-induction in high density shaking cultures.. Protein Expr Purif.

[38] Zhang WW, Matlashewski G (2000). Analysis of antisense and double stranded RNA downregulation of A2 protein expression in Leishmania donovani.. Mol Biochem Parasitol.

[39] Ma S (2000). Identification and characterisation of HASPB homologues in New World *Leishmania* species..

[40] Pimenta PF, Pinto da Silva P, Rangarajan D, Smith DF, Sacks DL (1994). Leishmania major: association of the differentially expressed gene B protein and the surface lipophosphoglycan as revealed by membrane capping.. Exp Parasitol.

[41] Sadlova J, Price HP, Smith BA, Votypka J, Volf P (2010). The stage-regulated. HASP and SHERP proteins are essential for differentiation of the protozoan parasite, *Leishmania major*, in its sand fly vector, *Phlebotomus papatasi*.. Cellular Microbiology, Jul.

[42] Requena JM, Soto M, Quijada L, Alonso C (1997). Genes and chromosomes of Leishmania infantum.. Mem Inst Oswaldo Cruz.

[43] Smith M, Blanchette M, Papadopoulou B (2008). Improving the prediction of mRNA extremities in the parasitic protozoan Leishmania.. BMC Bioinformatics.

