# Supplementary figures and images for: Leishmania-Specific Surface Antigens Show Sub-Genus Sequence Variation and Immune Recognition
Source: PLoS Negl Trop Dis. 2010 Sep 28;4(9):e829. doi: 10.1371/journal.pntd.0000829 (PMC2946902; doi:10.1371/journal.pntd.0000829)

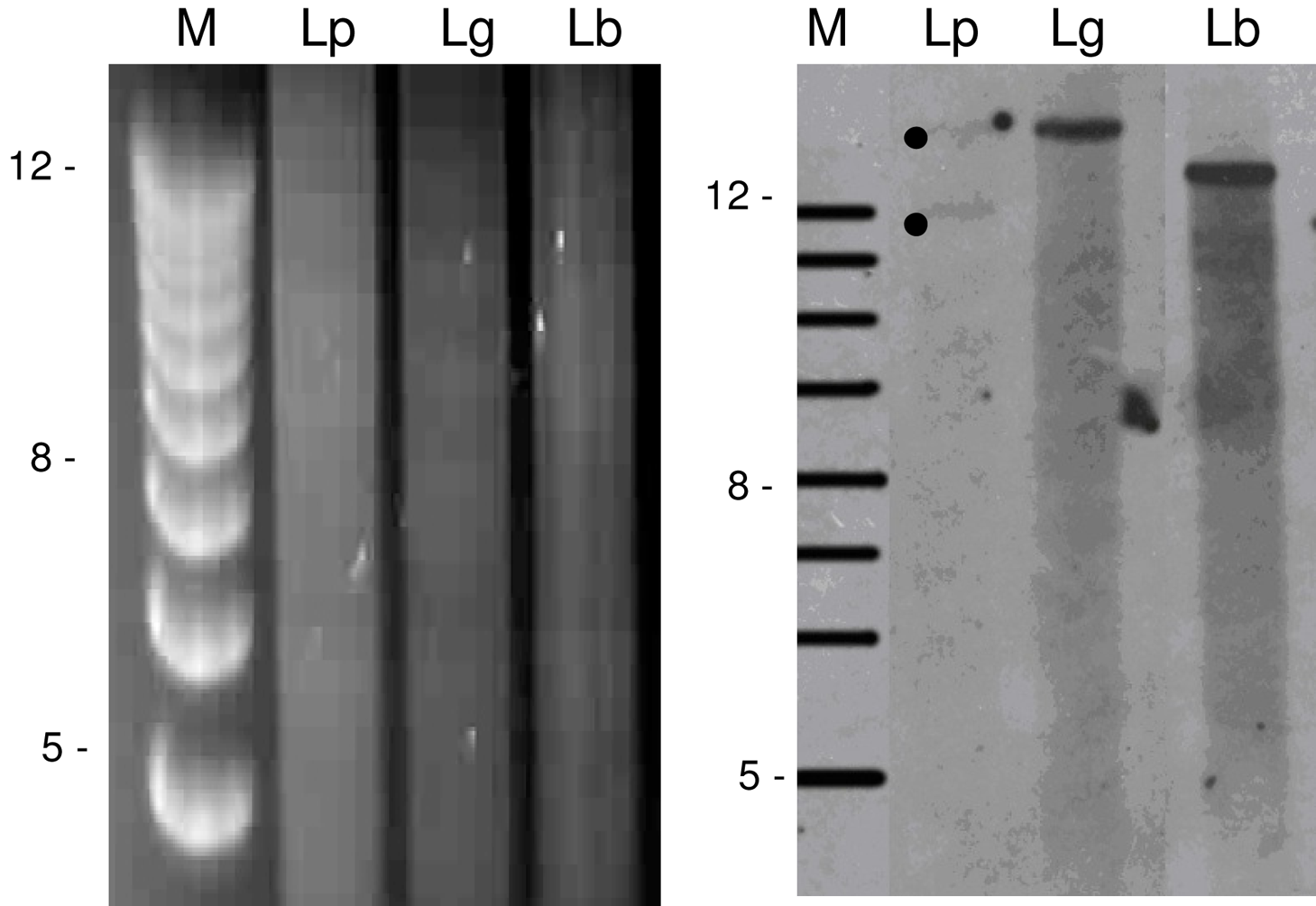

Supplement: Figure S1 — DNA hybridization analysis indicating the relative size of the OHL locus in L. Viannia species. 250 ng of genomic DNA from L. (V.) peruviana (Lp), L. (V.) guyanensis (Lg) and L. (V.) braziliensis (Lb), extracted from strains listed in Table 1 (Lb from strain M290475) were digested with XhoI and HinDIII, size separated through 0.6% agarose and hybridized with a digoxigenin probe targeting a repetitive intergenic region (vertical black bars in Figure 2B). A single hybridizing band was observed in the L. (V.) guyanensis and L. (V.) braziliensis digests while two weaker bands (black dots) were detected for L. peruviana. Molecular markers (M) are shown on the left (Kb). (0.76 MB TIF) [file pntd.0000829.s001.tif]

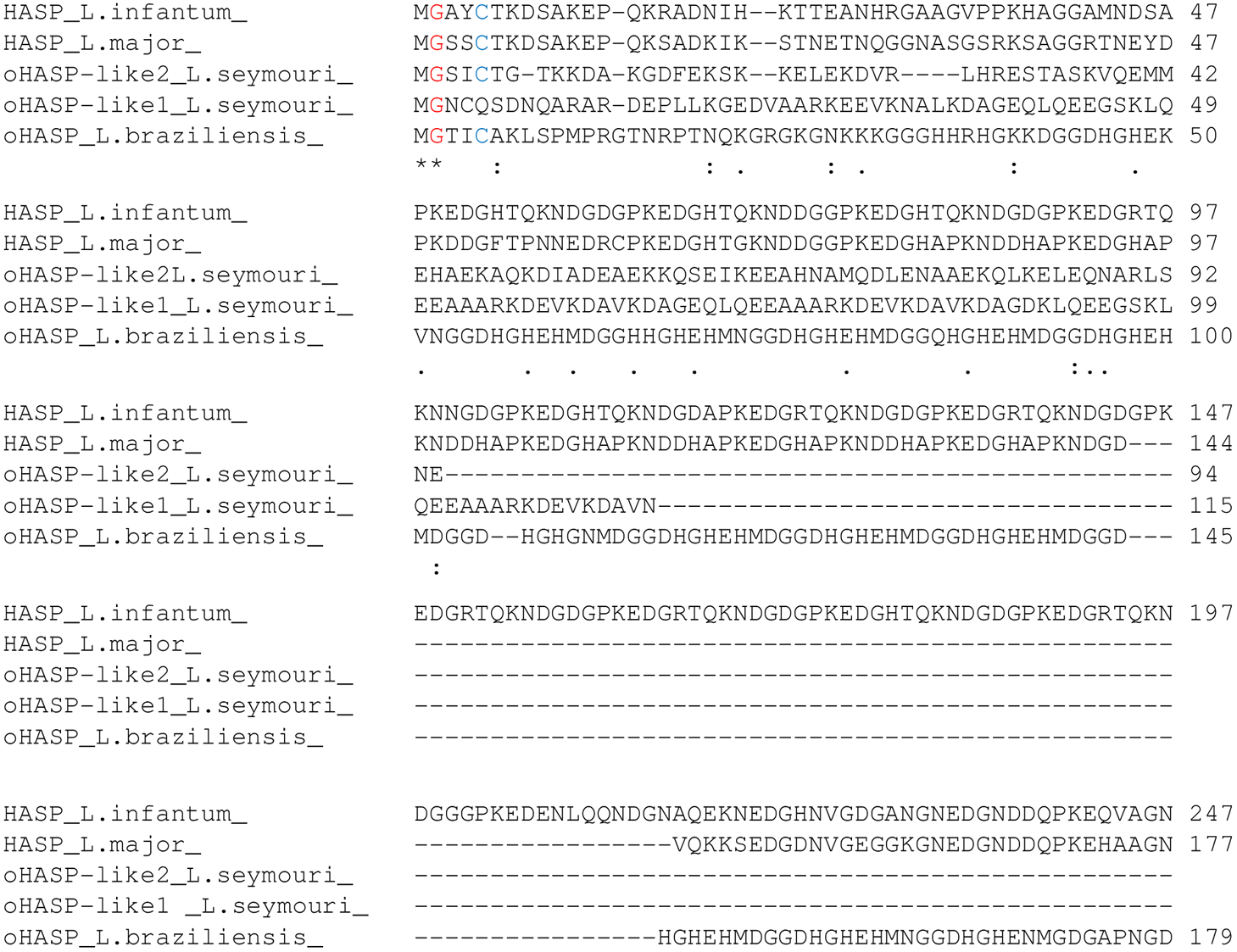

Supplement: Figure S2 — CLUSTALW alignment of the translated oHASP ORF (Lb1110, containing 14 repeat units) with HASPB sequences from L. (L.) major, L. (L.) infantum and the translated orthologous ORF identified in L. seymouri. N-myristoylation and palmitoylation sites are shown highlighted in red and blue respectively; conserved residue(*); conserved substitutions(:); semi-conserved substitution (.) (0.47 MB TIF) [file pntd.0000829.s002.tif]

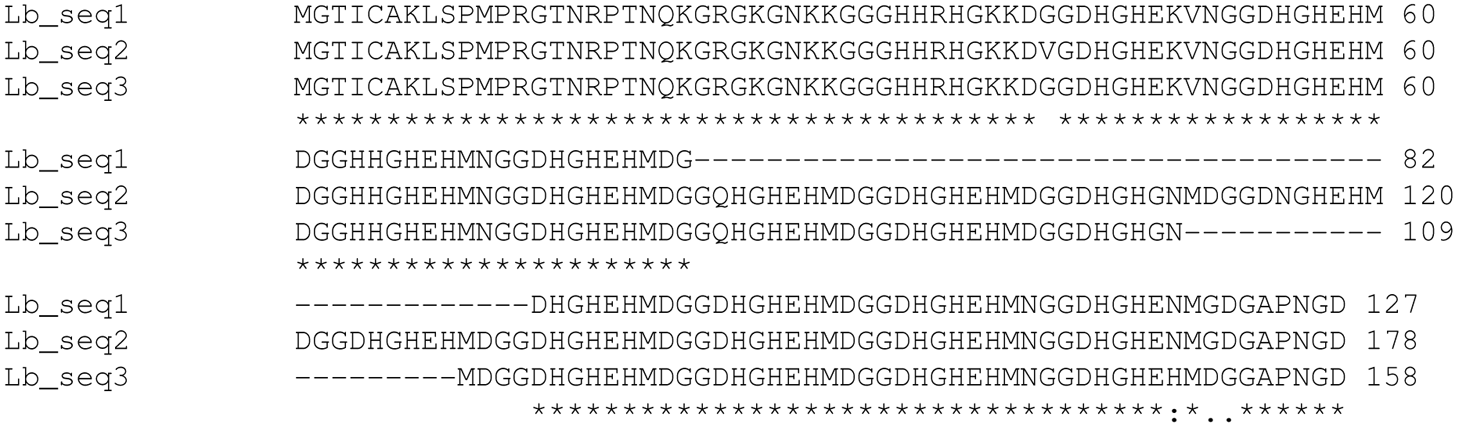

Supplement: Figure S3 — CLUSTALW alignment of the sequenced OHL ORFs (containing 9, 13 and 14 repeat units) revealing variation in both the sequence and number of repeated motifs in the amino acid repeat domains. (0.25 MB TIF) [file pntd.0000829.s003.tif]

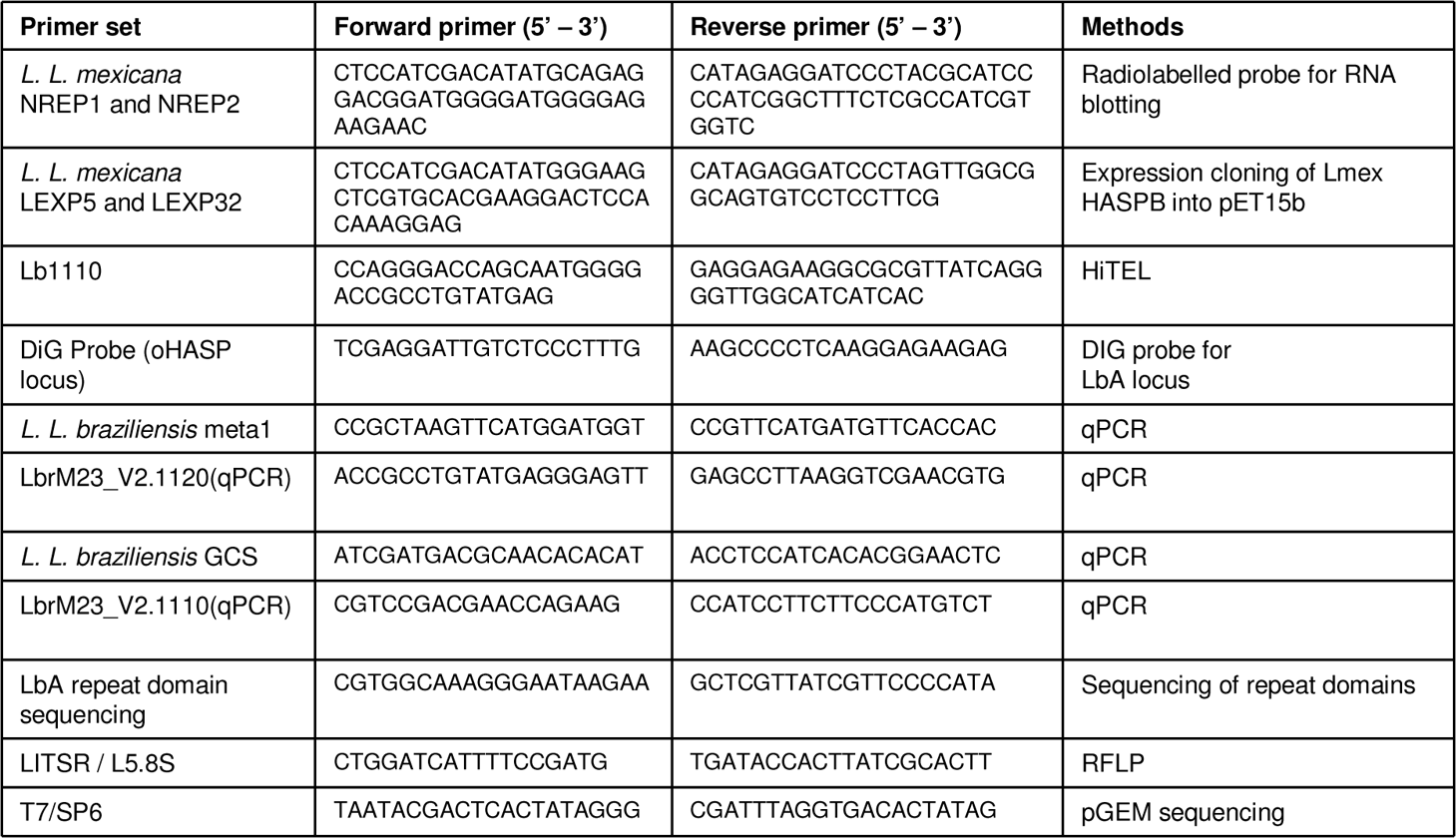

Supplement: Table S1 — Primers used for PCR amplifications in this study. (0.35 MB TIF) [file pntd.0000829.s004.tif]
